# Comparative Study of Externalized Ureteral Catheter Versus Double-J Stent on Percutaneous Nephrolithotomy: A Randomized Controlled Trial

**DOI:** 10.7759/cureus.22967

**Published:** 2022-03-08

**Authors:** Bilal Habib, Sadiqa Hassan, Mohammad Roman, Khursheed Anwar, Amber Latif

**Affiliations:** 1 Department of Urology, Pakistan Atomic Energy Commission (PAEC) General Hospital, Islamabad, PAK; 2 Department of Internal Medicine, Khan Research Laboratories (KRL) Hospital, Islamabad, PAK

**Keywords:** double-j stent, renal calculi, percutaneous nephrolithotomy (pcnl), nephrolithiasis, external ureteral catheter

## Abstract

Background: External ureteral catheter (EUC) and double-J stent are both commonly used to drain upper urinary tract in percutaneous nephrolithotomy (PCNL). We compared the outcomes of using EUC versus double-J stent in performing PCNL in patients with renal stones in our settings in order to identify a better technique for the management of renal stones in terms of postoperative stent-related symptoms.

Methods: This randomized controlled trial was conducted at the Department of Urology, PAEC General Hospital, Islamabad, from January 2020 to December 2020. A total of 80 patients of either gender between ages 18 and 70 years planned for PCNL were enrolled and randomized into group I (double-J stent) and group II (EUC). Outcomes of the procedure were compared in both groups.

Results: There were 62.5% of patients in group I who demonstrated stent-related symptoms compared to 22.5% in group II (p=0.001). No statistically significant difference was noted in other outcome variables like urinary leak (10% vs. 20%, p=0.210), post-procedure fever (25% vs. 22.5%, p=0.793), mean analgesia requirement (60.8 mg vs. 58.5 mg, p=0.685), and mean length of hospital stay (3.9 days vs. 4.2 days, p=0.330).

Conclusion: Stent-related symptoms were demonstrated by a significantly lesser number of patients who underwent PCNL with EUC when compared with patients who underwent PCNL with double-J stent. For other outcome variables (urinary leak, post-procedure fever, mean analgesia requirement, and mean length of hospital stay ), no significant difference was noted among both the groups.

## Introduction

Renal calculus disease is the third most common problem of urinary tract after urinary tract infection and prostatic pathologies [1]. The worldwide prevalence, incidence, and composition of calculi vary and in Asia, 1-19.1% of the population suffers from urolithiasis [2,3]. With the advances of surgical technology, less invasive procedures such as percutaneous nephrolithotomy (PCNL) have gradually become a preferred therapy for urinary stones [4,5]. Since its introduction, a variety of alterations and improvements has been made to the procedure in order to achieve maximum stone clearance along with decreasing the morbidity in patients, postoperative analgesic requirement, and postoperative hospital stay [6,7].

External ureteral catheter (EUC) and double-J stent are both commonly used to drain upper urinary tract in PCNL. A number of studies have confirmed the safety and efficacy of PCNL with double-J stent [8,9]. Double-J insertion is associated with number of urinary symptoms, flank, and suprapubic pain as well as urinary tract infections (UTIs) [10]. Zhou et al. in a recent study compared the outcomes of using externalized ureteral catheter versus double-J ureteral stent in tubeless minimally invasive percutaneous nephrolithotomy. Their result showed that stent-related symptoms were reported in 19.6% of patients in the EUC group while they were reported in 60.4% of patients in the double-J stenting group (p=0.001). No significant difference was noted in pain score (visual analog scale {VAS}: 2.80±2.49 vs. 2.92±2.07, p=0.408), urinary extravasation (7.1% vs. 9.4%. p=0.931), and length of hospital stay (5.70±2.72 days vs. 5.72±2.08 days, p=0.961) in both the groups [11]. Joshi et al. in another similar study evaluated the outcome of standard PCNL using two different stenting techniques, i.e., externalized ureteral catheter placement compared with double-J stent placement and reported that 64% of patients showed stent-related symptoms in the double-J stenting group, while in the EUC group, 28% of patients experienced such complications [12].

External ureteral catheter and double-J stent are both commonly used to drain upper urinary tract in PCNL. Double-J (DJ) insertion during PCNL is associated with number of adverse effects, which impact the quality of life and take a longer duration to return to their normal daily life activities. Furthermore, the DJ stent has to be removed after a number of days, which causes further distress and incurs extra cost. Externalized ureteral catheters, on the other hand, are associated with lower rates of postoperative symptoms, are easier to remove with no additional distress to the patient, and have no extra cost involved. The objective of our study was to compare these two techniques in our settings. The study results would help in identifying better techniques for the management of renal stones in terms of postoperative stent-related symptoms.

## Materials and methods

This randomized controlled trial was conducted at the Department of Urology, PAEC General Hospital, Islamabad, from January 2020 to December 2020. After the approval of the ethical committee of the PAEC General Hospital (study protocol code number: PGHI-IRB(DMe)-RCD-06-002), Islamabad, a total of 80 patients of either gender between the ages of 18 and 70 years were enrolled in this study, where the patients with cumulative stone diameter ≤ 4 cm, without ureteral obstruction, and with only a single access site were planned for PCNL. Patients with bleeding disorders, presence of significant residual calculi, pyuria, perforation of the renal collecting system, severe intraoperative or postoperative hemorrhage and second-look procedure necessity were excluded from this study.

Enrolled patients were equally randomized into two groups (40 in each) by lottery method. Patients of group I were placed with double-J stent and in group II patients, external ureteral catheter (EUC) were placed. Prophylactic antibiotics were given to all the enrolled individuals 30 minutes prior to the procedure. Then all the enrolled patients underwent PCNL procedure by the surgeon as per the standard protocol of our setting. In group I, DJ stent was placed anterogradely under fluoroscopy. In group II, EUC was left at end of the procedure. The EUC was removed on the day of discharge while DJ stent was left in place for two to four weeks and was removed as an outpatient under local anesthesia. All preoperative, intraoperative, and postoperative data were recorded for each patient.

Outcomes of the procedure were compared in both groups in terms of stent-related symptoms (assessed on postoperative day one, day two, and two weeks), postoperative fever, urinary leak during hospital stay (extravasation of urine at the incision site postoperatively), total dose of analgesia required during hospital stay (a dose of analgesia {IV Toradol in a dose of 30mg} was given if VAS score was ≥4) and length of hospital stay. After two weeks, a follow-up evaluation to judge stent-related symptoms was done. 

Collected data were entered and analyzed by using statistical software SPSS version 22.0 (Armonk, NY: IBM Corp.). Outcomes were compared in both the groups using chi-square test for qualitative variables and independent sample t-test for quantitative variables. Effect modifiers like age, gender, size, and side of stone were stratified and a post-stratification chi-square test was applied to compare qualitative and independent sample t-test was applied to compare quantitative variables. A p ≤0.05 was considered as significant in all cases.

## Results

Characteristics of patients and diseases are shown in Table 1. The overall mean age of patients was 40.6±14.8 years with a male-to-female ratio of 1:1.1. The majority of patients (68.8%) belonged to the age group 18-50 years. The overall mean size of the stone was 2.2±0.64 cm and the majority of patients (57.5%) had large stone (>2cm) and on the left side (56.3%). Postoperative outcomes of externalized ureteral catheter and double-J stent in percutaneous nephrolithotomy are shown in Table 2.

**Table 1:** Characteristics of patients and disease (n=80)

Variables		Group I (n=40)	Group II (n=40)
Age (years)	Mean±SD	39.7±11.7	41.5±17.5
	Range	18-64	21-70
Gender	Male	21 (52.5%)	17 (42.5%)
	Female	19 (47.5%)	23 (42.5%)
Stone size (cm)	Mean±SD	2.4±0.65	1.9±0.40
Stone side	Right kidney	17 (42.5%)	18 (45.0%)
	Left kidney	23 (57.5%)	22 (55.0%)

**Table 2:** Postoperative outcome of externalized ureteral catheter and double-J stent in percutaneous nephrolithotomy *Chi-square test. **The value is significant. ***The value is not significant.

Outcomes	Group I (n=40)	Group II (n=40)	Overall (n=80)	p-Value*
Stent-related symptoms (%)	25 (62.5%)	9 (22.5%)	34 (42.5%)	0.001**
Urinary leak (%)	4 (10.0%)	8 (20.0%)	12 (15.0%)	0.210***
Fever (%)	10 (25.0%)	9 (22.5%)	19 (23.8%)	0.793***
Analgesic requirement (mg) (mean±SD)	60.8±20.9	58.5±27.9	59.2±23.5	0.685***
Mean length of hospital stay (mean±SD)	3.9±0.92	4.2±1.71	4.1±0.89	0.330***

## Discussion

With the advancement of surgical technology, less invasive procedures such as percutaneous nephrolithotomy (PCNL) have gradually become a preferred therapy for urinary stones [13]. Since its introduction, a variety of alterations and improvements has been made to the procedure to achieve maximum stone clearance while also decreasing the morbidity in patients [6]. Some of these alterations are the use of a nephroscope with smaller outer sheath, tubeless PCNL, total tubeless PCNL, injecting a hemostatic agent in the nephrostomy tract, and performing PCNL on outdoor basis under local anesthesia [7]. In the tubeless technique, both the EUC and double-J stent are commonly used to drain upper urinary tract [10].

Our results showed that 62.5% of patients in group I demonstrated stent-related symptoms compared to 22.5% in group II (p=0.001). No statistically significant difference was noted in other outcome variables like urinary leak (10% vs. 20%, p=0.210), post-procedure fever (25% vs. 22.5%, p=0.793), mean analgesia requirement (60.8±20.9mg vs. 58.5±27.9mg, p=0.685), and mean length of hospital stay (3.9±0.92 vs. 4.2±1.71 days, p=0.330) in our study. Shah et al. found that 30% of the patients experienced discomfort related to DJ stent placement [14]. Similarly, 52.1% of the patients had some sort of stent-related symptom in a study by Gonen et al. This indicated that other discomforts in addition to pain were caused by DJ positioning. Gonen et al. reported that using EUC instead of DJ stent for postoperative drainage did not increase postoperative morbidity of the tubeless PCNL [15].

Mouracade et al. concluded that the replacement of DJ with EUC in tubeless PCNL was a safe and effective procedure for patients with a mean stone burden of 17.25 mm [16]. Similarly, our results also showed that replacing the DJ with EUC for postoperative drainage reduced the stent-related discomfort without increased morbidity of tubeless PCNL.

Similar to our study, Zhou et al. in a recent study compared the outcomes of using externalized ureteral catheter versus double-J ureteral stent in tubeless minimally invasive percutaneous nephrolithotomy and showed that stent-related symptoms were reported in 19.6% of patients in the EUC group while they were reported in 60.4% of patients in double-J stenting group (p=0.001). No significant difference was noted in pain score (VAS: 2.80±2.49 vs. 2.92±2.07, p=0.408), urinary extravasation (7.1% vs. 9.4%, p=0.931), and length of hospital stay (5.70± 2.72 vs. 5.72±2.08, p=0.961) in both the groups [11].

Chen et al. compared the efficacy and safety of EUC and DJS in tubeless PCNL and revealed that stent-related symptoms were higher for DJ stent compared with EUC (odds ratio {OR}: 0.09; 95% confidence interval {CI}: 0.01-0.61; p=0.01). No significant differences were found in analgesic required (OR: 1.02; 95% CI: 0.77-1.34; p=0.91), stone-free rate (risk ratio: 0.98; 95% CI: 0.9-1.07; p=0.67), and duration of hospitalization (weighted mean difference {WMD}: -0.21 days; 95% CI: -0.86 to 0.44; p=0.53) [17]. Our results are similar to the study results by Chen et al., however, we did not measure the operative time and stone free rate in our study [17].

Compared with DJ stent, EUC is also an effective alternative for patients with upper urinary stones in tubeless PCNL and could help patients by reducing stent-related discomfort and avoiding cystoscopy for DJ stent removal. However, EUC will not replace DJ for patients with large stone size or large residual stone fragments. In our opinion, the choice of drainage technique should be individualized and careful selection of patients is one of the key factors for increasing the success rates and avoiding complications. We recommend further well-designed RCTs with larger sample size to validate our findings.

The limitations of our study were its small sample size and short follow-up period. The small sample size reduces the statistical power of our study and limits the ability to accurately ascertain the effect of our intervention. Further clinical trials with a longer follow-up period by using different individualized draining techniques are warranted to compare the externalized ureteral catheter versus double-J stent on percutaneous nephrolithotomy.

## Conclusions

Stent-related symptoms were demonstrated by significantly lesser number of patients who underwent percutaneous nephrolithotomy (PCNL) with externalized ureteral catheter (EUC) when compared with patients who underwent PCNL with double-J stent. For other outcome variables (urinary leak, post-procedure fever, mean analgesia requirement, and mean length of hospital stay ), no significant difference was noted among both the groups.
